# Weight trajectories after last tirzepatide or semaglutide prescription across a federated health network

**DOI:** 10.1093/biomethods/bpag020

**Published:** 2026-04-20

**Authors:** Karthik Murugadoss, Gowtham Varma, A J Venkatakrishnan, C Michael Gibson, Venky Soundararajan

**Affiliations:** nference, Cambridge, MA 02142, United States; Metabolism Agentic Intelligence Atlas (MAIA), Cambridge, MA 02142, United States; nference Labs, Bangalore, Karnataka 560017, India; nference, Cambridge, MA 02142, United States; Metabolism Agentic Intelligence Atlas (MAIA), Cambridge, MA 02142, United States; Baim Institute for Clinical Research, Harvard Medical School, Harvard University, Boston, MA 02215, United States; nference, Cambridge, MA 02142, United States; Metabolism Agentic Intelligence Atlas (MAIA), Cambridge, MA 02142, United States; nference Labs, Bangalore, Karnataka 560017, India

**Keywords:** GLP-1 receptor agonists, obesity, weight change, semaglutide, tirzepatide

## Abstract

GLP-1 receptor agonist (GLP-1RA) discontinuation has been associated with weight regain. However, weight trajectories following the last documented GLP-1RA prescription in the real-world clinical setting have not been explored. Here, we assessed weight trajectories of 4182 patients in the 6 months following their last semaglutide or tirzepatide prescription. Approximately two-thirds of patients showed stable weight or continued weight loss during this period. In a representative subset of 300 patients whose clinical notes were curated using a large language model, treatment discontinuation was documented for 119 patients (40%) around the time of the last prescription. Among these 119 patients, a similar pattern of weight trajectories was observed, with 72% of patients not demonstrating weight regain. Exercise counseling was documented more frequently among patients with durable weight loss after the last GLP1-RA prescription than among those with weight regain (26.2% vs. 14.7%; *P* = .04). Further studies are warranted to evaluate the mechanisms underlying these real-world patterns.

## Introduction

GLP-1 receptor agonists (GLP-1RAs) such as semaglutide and tirzepatide have transformed obesity care and weight management, delivering unprecedented levels of weight loss in clinical trials [1]. For example, in the phase 3 STEP-1 trial, patients receiving once-weekly dosing of 2.4 mg semaglutide plus lifestyle intervention had an average of 15% weight loss over a 68-week period, compared to only 2.4% for patients receiving placebo and lifestyle intervention [2]. In the phase 3 SURMOUNT-1 trial, once-weekly treatment with 15 mg of tirzepatide plus lifestyle intervention resulted in 21% weight loss over a 72-week period, compared to 3% for placebo [3]. These significant impacts on weight management have been corroborated in analyses of real-world data, for example, in a comparative analysis of semaglutide and tirzepatide which showed average weight loss of 8% and 15% over a 1-year period, respectively [4]. Multiple randomized and real-world studies have also shown that treatment with semaglutide or tirzepatide more broadly improves cardiovascular health, including reductions in rates of cardiovascular events such as acute myocardial infarction and stroke [5–7].

Despite these benefits, real-world studies have shown that more than half of patients discontinue GLP-1RAs within one year of initiation [8]. There are various cited reasons for discontinuation, including gastrointestinal adverse effects, lack of treatment effect, out-of-pocket costs, and supply chain shortages [9–11]. Several studies have demonstrated reversal of weight loss and other cardiometabolic benefits after patients discontinue GLP-1RAs [12–14]. For example, in an extension of the STEP-1 trial, patients regained approximately two-thirds of their lost weight in the year following discontinuation of semaglutide, and cardiometabolic parameters such as blood pressure and hemoglobin A1c reverted to near pre-treatment levels over the same time period [13]. Similarly, in a post-hoc analysis of the SURMOUNT-4 trial, most patients regained weight after discontinuation of tirzepatide, and the degree of weight regain was associated with the degree of reversion of cardiometabolic parameters, including blood pressure, cholesterol levels, and hemoglobin A1c [14]. A systematic review of 18 randomized clinical trials came to similar conclusions regarding “metabolic rebound” following GLP-1RA discontinuation [12]. To date, it is not clear whether there are subsets of patients who are more likely to experience continued benefit after their therapeutic exposure.

While the impact of discontinuation in a controlled setting (i.e. as part of a clinical trial protocol) is important to understand, it does not offer a comprehensive understanding of the true patient experience. Further, it fails to capture the perspective of the prescribing clinicians, whose patients may discontinue GLP-1RA therapy in line with or against their advice, transfer care to another provider within the same health system or be lost to follow-up for myriad reasons. This perspective has recently been even further complicated by the trend for patients to obtain GLP-1RA prescriptions from other sources, such as online providers or medical spas, with or without the knowledge of their physicians [15]. Thus, at the intersection of the patient and clinician experience, it is important to better understand weight trajectories following the last GLP-1RA prescription by a primary care or specialist provider. Here, we leverage the nSights platform to directly address this question using a large-scale longitudinal real-world dataset.

## Methods

### Study design and patient population

This was a retrospective observational study to assess patterns of weight change (“weight trajectories”) following the last GLP-1RA prescription by a provider within a federated system of multiple health networks. Patients were eligible for inclusion in the study if they had (i) at least three prescriptions for semaglutide or tirzepatide at most 4 months apart within 1-year period between 08 January 2019 to 07 January 2025, (ii) subsequently had at least 6 months without a prescription for any GLP-1RA medication, and (iii) had at least one weight measurement during their on-therapy time and at least one weight measurement within six months after the date of their last GLP-1RA prescription. Patients were excluded if they ever had a prescription for another GLP-1RA therapy, in order to limit the potential of confounding due to treatment-switching effects. For each patient, the index date was set as the date of their last GLP-1RA prescription, specifically the date of the GLP-1RA prescription that was followed by at least 6 months without another prescription for the same medication. Semaglutide and tirzepatide were analyzed separately to account for their distinct mechanistic properties as a GLP-1 receptor agonist and dual GLP-1/GIP receptor agonist, respectively.

### Analysis of weight trajectories

Weight measurements were analyzed from 12 months before the last prescription through 6 months after the last prescription (−365 to +180 days from the index date). A 6-month follow-up window was selected to ensure adequate availability of follow-up weight measurements across health systems while minimizing attrition bias inherent in real-world EHR data. Baseline weight is defined as the weight measurement closest to the last prescription date within ±90-day window. For each measurement, percentage weight change was calculated as the difference from baseline weight divided by baseline weight, multiplied by 100. Measurements were then grouped into 30-day bins relative to the index date. Patients were grouped retrospectively based on their post-last prescription trajectories into two clinically intuitive categories: (1) patients with at least 2% increase in weight in the six months following their last GLP-1RA prescription (“weight regain”); (2) patients without at least 2% increase in weight following their last GLP-1RA prescription (“non-regain”). Of note, a 2% increase was selected as a pragmatic threshold to distinguish meaningful weight changes from normal short-term variability in body weight measurements, although this specific threshold does not have any intrinsic clinical significance.

To characterize cohort-level weight trajectories while accounting for variable measurement frequency across patients, a two-stage aggregation approach was employed. First, patient-level median weight change was calculated within each time bin to generate at most a single representative value per patient per bin. Cohort-level statistics, including mean and standard deviation, were then derived from these patient-level values. Ninety-five percent confidence intervals were computed as mean ± 1.96 × standard error of the mean, where standard error of the mean was defined as the standard deviation divided by the square root of the number of patients contributing to each bin.

### Definition of recommended maintenance doses

We evaluated maintenance dose attainment within the 12 months preceding (and including) the date of last prescription. Maintenance doses were defined as the minimum labeled therapeutic dose for each formulation: 0.5 mg for Ozempic, 1.7 mg for Wegovy, 7 mg for Rybelsus, and 5 mg for Mounjaro and Zepbound. A patient was classified as having attained maintenance dosing if at least one prescription during this window met or exceeded the threshold for their corresponding medication. The percentage of patients reaching maintenance dose was calculated separately for semaglutide and tirzepatide, with the denominator comprising all patients with sufficient follow-up data for trajectory classification.

### Content extraction from clinical notes

We applied a large language model (LLM) with schema-constrained structured decoding to extract GLP-1 treatment context from longitudinal clinical notes for all study patients at one of the academic medical centers (AMC) within the federated network. For each patient, we selected notes within 12 months before and after the index date (last prescription) that contained a generic or brand mention of semaglutide or tirzepatide. Extraction was performed using GPT-OSS 20B, deployed via vLLM (version 0.13.0) in Python (version 3.13), with an average input token length of 3.5k tokens per note. Inference was conducted with temperature 0.1 and top_*k* = 1 using a fixed random seed to ensure reproducibility. The LLM produced note-level JSON outputs capturing (i) clinical-documented GLP-1 status changes (e.g. held, stopped, restarted, switched) and dosing information, (ii) lifestyle counseling (diet and exercise), (iii) use of other anti-obesity medications, and (iv) optional context such as access barriers or outside sourcing when explicitly stated. All extracted events required short verbatim evidence excerpts from the note to support provenance and note-level extractions were subsequently aggregated into patient-level indicators relative to the index date for downstream cohort summaries and comparisons across outcome groups.

### Statistical analysis

Differences in the frequency of lifestyle counseling documentation between patients with and without weight regain were evaluated using chi-square tests for categorical variables. Two-sided *P*-values less than .05 were considered statistically significant. Analyses were performed separately for diet counseling and exercise counseling mentions extracted from clinical notes documented after the last GLP-1RA prescription. All statistical analyses were conducted using Python (version 3.13) with the SciPy library (version 1.15.1).

## Data source and selection

This study analyzed de-identified EHR data from a network of tertiary clinical centers tied to AMC in the USA through the nference nSights Analytics Platform. nference, in collaboration with AMC data partners, provided the de-identified data for this study. nference has established a secure data environment, hosted by and within each of the AMCs, that contains the AMC’s de-identified patient data. The provisioning of and access to this data are governed by an expert determination that satisfies the Health Insurance Portability and Accountability Act (HIPAA) Privacy Rule requirements for the de-identification of protected health information. Each AMC’s de-identified data environment is specifically designed and operated to enable access to and analysis of de-identified data without the need for Institutional Review Board (IRB) oversight, approval, or an exemption confirmation. Given these measures, informed consent and IRB review were not required for this study.

## Code availability

The code used in this study is not publicly available. Requests for additional information should be directed to the corresponding author.

## De-identification and HIPAA compliance certification

Prior to analysis, all EHR data were de-identified under an expert determination consistent with the HIPAA Privacy Rule (45 CFR §164.514(b)(1)). The de-identification methodology employed a multi-layered transformation approach to both structured and unstructured data fields [16, 17]. In structured data, direct identifiers including patient names and precise geographic locations were excluded entirely, while indirect identifiers underwent specific transformations: patient identifiers, medical record numbers, and accession numbers were replaced with one-way cryptographic hashes using confidential salts to preserve linkage across patient encounters; all dates were shifted backward by patient-specific random offsets (1–31 days) to preserve temporal relationships while obscuring exact event timing; the ZIP codes were truncated to two-digit state-level resolution; and continuous variables including age, height, weight, and body mass index were thresholded to prevent identification of extreme values (for example, ages ≥89 years transformed to ‘89+’ and BMI >40 transformed to ‘40+’). In unstructured clinical text, an ensemble de-identification system that combines attention-based deep learning models with rule-based methods achieved an estimated >99% recall for personally identifiable information detection, with detected identifiers replaced by plausible fictional surrogates [16].

## Data harmonization

To address heterogeneity in EHR data, we harmonized clinical variables, including medications, anthropometric measurements, and diagnoses, to standardized concepts. For medications, we first constructed a standardized drug concept database combining the nSights knowledge graph with RXNorm (https://www.nlm.nih.gov/research/umls/rxnorm/index.html) hierarchies to capture ingredient, brand, and dose-specific information [18]. EHR medication records were matched using a hierarchical approach prioritizing RXNorm codes when available, followed by ingredient-level matching, and finally natural language processing and pattern matching on free-text medication orders when structured codes were absent. For anthropometric measurements (height, weight, BMI), we created a unified vocabulary from SNOMED (https://www.snomed.org/, https://athena.ohdsi.org) and LOINC (https://loinc.org/) terminologies and matched EHR measurement descriptions using standardized text matching algorithms with abbreviation expansion and synonym resolution; ambiguous mappings were resolved using OpenAI GPT-4o (https://platform.openai.com/docs/models/gpt-4o) with summary statistics as context, followed by manual verification. For diagnoses, we developed a hierarchical disease concept database from the nSights knowledge graph and matched EHR diagnosis descriptions and codes by identifying the most specific common child concept in the hierarchy. This approach enabled consistent identification of clinical entities while preserving granularity where available.

## Results

There were 2567 patients in the semaglutide cohort and 1615 patients in the tirzepatide cohort. Demographic and baseline clinical characteristics are summarized in Table 1. Briefly, the semaglutide cohort was about 71.2% female and 77.3% white with a mean age of 55 years (SD: 13 years); the tirzepatide cohort was about 70.2% female, 85.3% white, and had a mean age of 55 years (SD: 12 years). At the time of the last prescription, the prevalence of type 2 diabetes and obesity, respectively, were 60.0% and 66.0% in the semaglutide cohort, and 77.0% and 82.5% in the tirzepatide cohort. The average baseline weight (i.e. approximately 12 months before the last prescription) in the semaglutide and tirzepatide cohorts was 103.3 and 108.5 kg, respectively, and average BMI was 34.6 and 35.5 kg/m^2^, respectively. At least one prescription for a recommended maintenance dose (see the section “Methods”) during the year prior to the last prescription was present for 90.6% of patients taking semaglutide and 95.3% of patients taking tirzepatide.

**Table 1:** Demographics and baseline clinical characteristics for the semaglutide and tirzepatide cohorts.

	Semaglutide cohort (*N* = 2567)	Tirzepatide (*N* = 1615)
**Age**		
**Age at last prescription (mean, std)**	55.3 (12.8)	55.1 (11.8)
**Sex**		
**Female (*N*, %)**	1828 (71.2%)	1134 (70.2%)
**Male (*N*, %)**	739 (28.8%)	481 (29.8%)
**Race**		
**White (*N*, %)**	1984 (77.3%)	1378 (85.3%)
**African American/Black (*N*, %)**	421 (16.4%)	131 (8.1%)
**Other (*N*, %)**	162 (6.3%)	106 (6.6%)
**Clinical characteristics**		
**Type 2 Diabetes mellitus (*N*, %)**	1540 (60.0%)	1066 (66.0%)
**Obesity (*N*, %)**	1977 (77.0%)	1332 (82.5%)
**Weight, kg**		
**Mean (SD)**	103.3 (22.6)	108.5 (22.8)
**Median [IQR]**	100.3 [87.2–117.1]	107 [91.3–123.8]
**BMI, kg/m^2^**		
**Mean (SD)**	34.8 (5.5)	35.5 (5.7)
**Median [IQR]**	34.6 [31.1–38.1]	35.5 [31.9–39.3]

**Figure 1:**
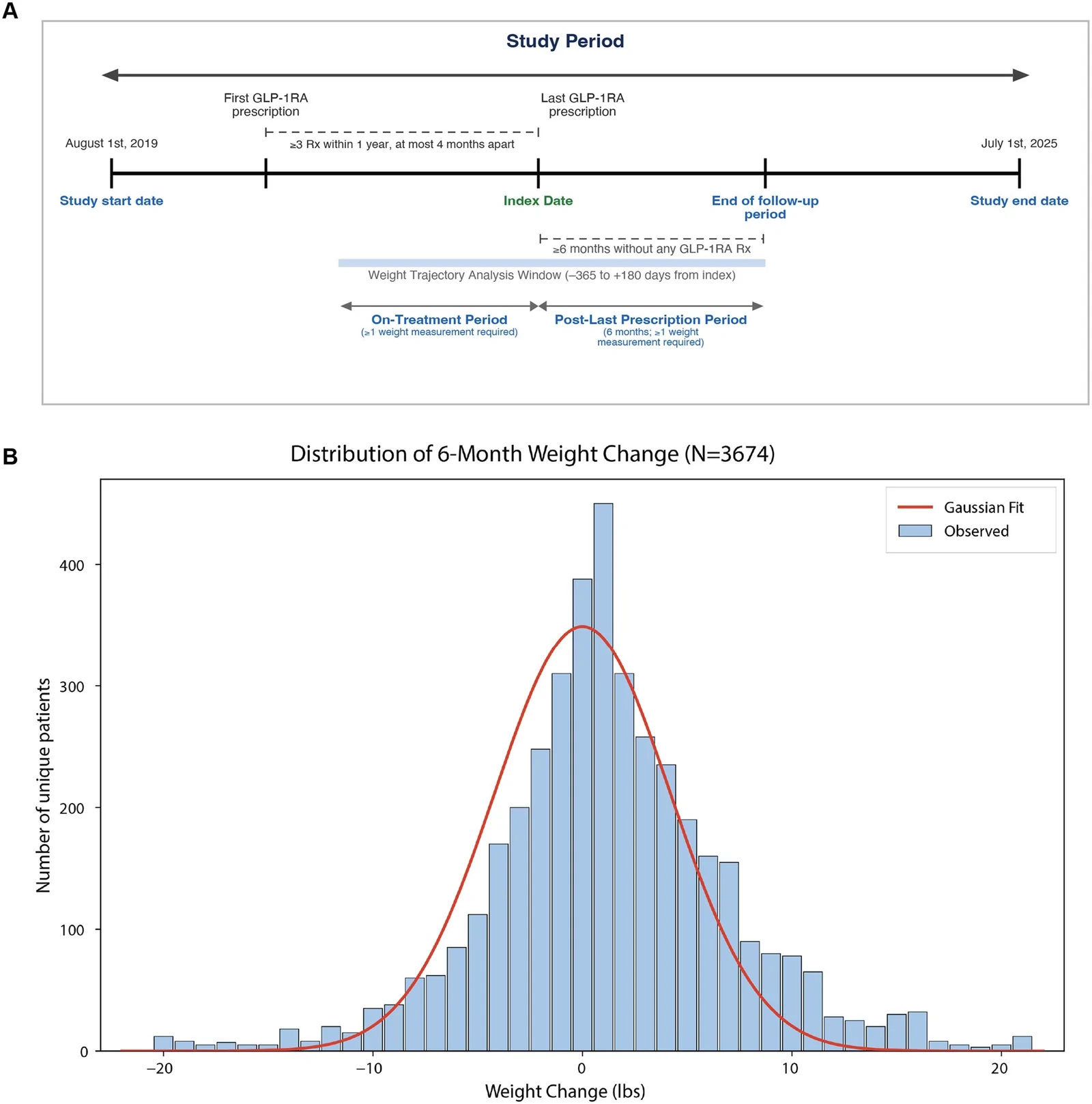
(A) Study schematic. Weight trajectories of patients with at least three prescriptions of semaglutide or tirzepatide were assessed for 6 months following the last prescription. (B) Histogram depicting the distribution of weight change during the follow-up period. For each individual patient, the weight measurements that were chronologically closest to the index date (date of late prescription) and 6 months after the index date were used to calculate the patient-level weight change.

**Figure 2:**
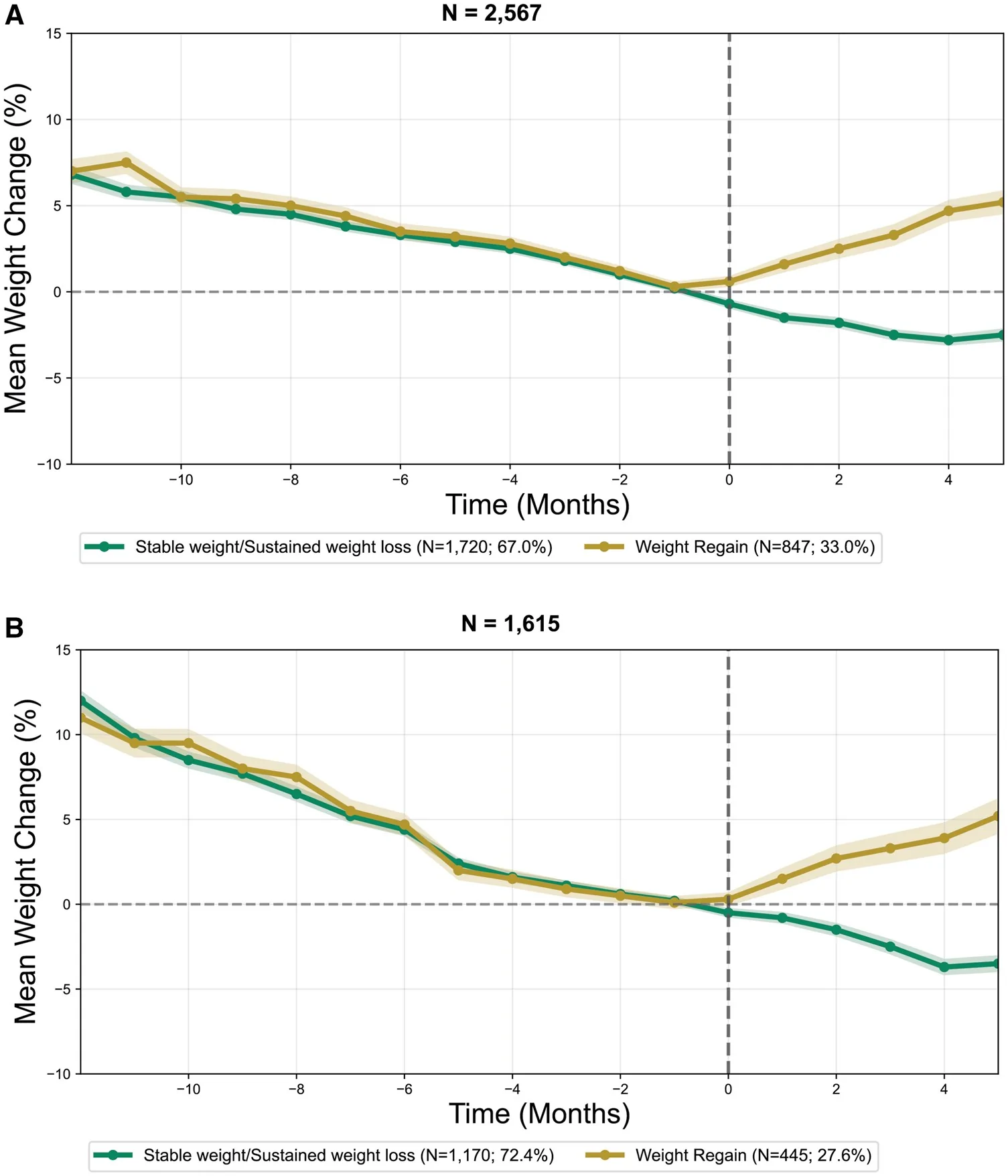
Weight loss trajectories of patients leading up to and after the last prescription of semaglutide (A) or tirzepatide (B), divided into subsets of patients who demonstrated weight regain (orange) versus stable weight or continued weight loss (green).

Among these cohorts, we assessed the average percent change in weight in the year leading up to the last prescription (defined as month 0) and in the 6 months following the last prescription (Fig. 1A). The patient-level weight change after the last GLP-1RA prescription is approximately normally distributed with a mean value of 0.29% (median value of 0), indicating that most patients did not experience significant weight gain following their last GLP-1RA prescription (Fig. 1B). Patients were grouped retrospectively based on their post-last prescription trajectories into two clinically intuitive categories based on whether their weight increased by at least 2% in the 6 months following their last GLP-1RA prescription, labeled as “weight regain” and “non-regain” groups (see the section “Methods”). In both the tirzepatide- and semaglutide-treated cohorts, more than two-thirds of patients fell into the non-regain group (Fig. 2). Of note, the weight loss trends in the year leading up to the last prescription were quite similar between the weight regain and non-regain groups for both semaglutide (∼7% weight loss) and tirzepatide (∼11% weight loss). At the time of the last prescription, the weight and BMI distributions in the two groups were also similar for both medications (Fig,  3). Among patients who regained weight after their last GLP-1RA prescription (33.0% for semaglutide, 27.6% for tirzepatide), the average weight regain reached 4% within 4 months of the last prescription (Fig. 2).

**Figure 3:**
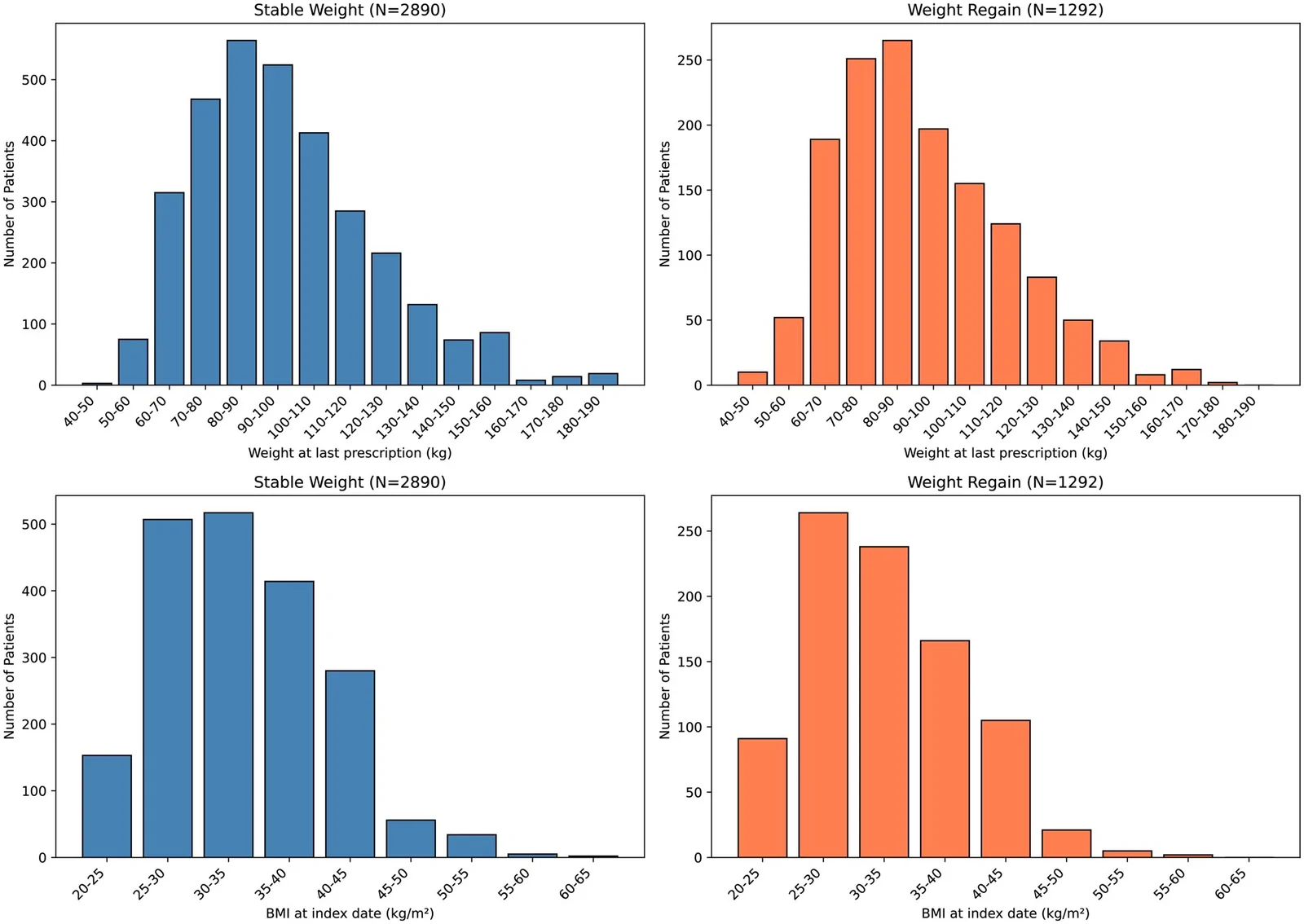
Weight (top row) and BMI distributions (bottom row) for the cohorts of patients with stable weight following the last GLP-1RA prescription (left column) or weight regain following the last prescription (right column). The weight and BMI closest to the date of the last prescription were selected for each patient.

**Figure 4:**
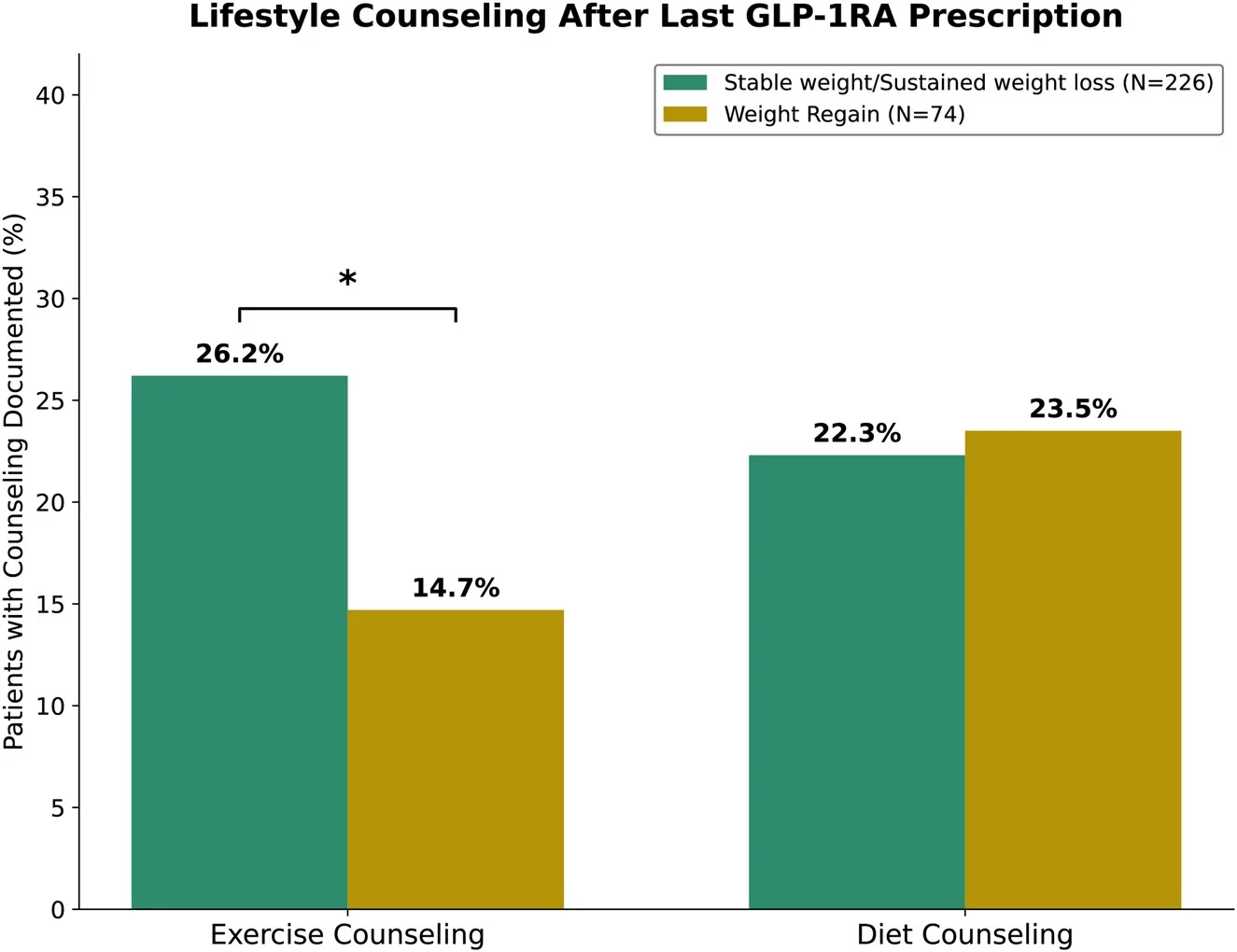
Comparison of rates of documented lifestyle counseling in the period following the last GLP-1RA prescription, including exercise counseling and diet counseling. All patients from a single health system within the federated network (*N* = 300) were included in this analysis. Statistical significance: *, *P* < .05.

We next evaluated whether lifestyle factors, specifically diet and physical activity, might be associated with sustained weight maintenance. At a representative site, we applied LLMs to analyze the frequency of lifestyle counseling documented in longitudinal EHR notes (see the section “Methods”) among patients who did not experience weight regain (*N* = 226) versus those who did (*N* = 74). Patients without weight regain were more likely to have exercise counseling mentions than those with regain (26.2% vs. 14.7%; *P* = .04; Fig. 4). In contrast, rates of diet counseling after the last prescription were similar between these groups (22.3% vs. 23.5%; *P* = .83; Fig. 4).

The analyses presented thus far are anchored on the date of each patient’s last prescription, which does not necessarily correspond to medication discontinuation. To better understand the relationship between these events, we deployed an LLM across clinical notes from a representative subset of 300 patients (159 taking semaglutide, 141 taking tirzepatide) to determine how frequently there was supporting clinician documentation of treatment cessation within ±90 days of the last prescription (see the section “Methods”). There was such documentation for 119 patients (40%) overall, including 68 of 159 (43%) semaglutide patients and 61 of 141 (36%) tirzepatide patients (Table 2). Among the 119 patients with clinician-documented GLP-1RA discontinuation, 86 (72%) were in the non-regain group, including 51 of 68 (75%) semaglutide patients and 35 of 51 (69%) tirzepatide patients (Table 2). It was rare to find notes indicating that patients subsequently obtained the same or another GLP-1RA from another source (Table 2), although this likely underestimates the true prevalence of such behavior due to incomplete reporting and documentation.

**Table 2:** Summary of LLM-extracted content from clinical notes in the analyzed cohorts, split by both medication (semaglutide versus tirzepatide) and weight trajectory in the 6 months following the last prescription (weight regain versus non-regain).

	Total	Semaglutide		Tirzepatide	
Measure	*N* = 300	Non-regain (*N* = 116)	Regain (*N* = 43)	Non-regain (*N* = 110)	Regain (*N* = 31)
**Post-period notes available**	277 (92.3%)	110 (94.8%)	41 (95.3%)	98 (89.1%)	28 (90.3%)
**Clinician-documented discontinuation (±90d)**	119 (39.7%)	51 (44.0%)	17 (39.5%)	35 (31.8%)	16 (51.6%)
**Restart/switch after last prescription (post60d)**	21 (7.0%)	11 (9.5%)	5 (11.6%)	5 (4.5%)	0 (0.0%)
**Outside-source GLP-1RA use documented (follow-up)**	4 (1.3%)	2 (1.7%)	0 (0.0%)	2 (1.8%)	0 (0.0%)
**Other anti-obesity medication documented (follow-up)**	14 (4.7%)	2 (1.7%)	5 (11.6%)	7 (6.4%)	0 (0.0%)
**Weight status documented in notes (follow-up)**	147 (49.0%)	68 (58.6%)	26 (60.5%)	39 (35.5%)	14 (45.2%)
**Max pre-index dose, median[Q1, Q3]**	N/A	2.0 [1.7, 2.4]	2.4 [1.85, 2.4]	15.0 [10.0, 15.0]	12.5 [7.5, 15.0]

All patients from a single health system within the federated network (*N* = 300) were included in this analysis.

No weight regain/regain: Weight trajectory classification in the follow-up period after the last GLP-1RA prescription.

Post-period notes available: Proportion of patients with at least one clinical note available in the follow-up period after the last GLP-1RA prescription.

Clinician-documented discontinuation (±90d): Evidence of treatment cessation documented in notes within ±90 days of the last prescription date.

Restart/switch (post60d): Evidence of GLP-1RA restart or switching to another GLP-1RA occurring >60 days after the last prescription.

Outside-source GLP-1RA use (follow-up): Documentation suggesting GLP-1RA use outside the captured health system after the last prescription.

Other anti-obesity medication (follow-up): Documentation of non–GLP-1RA anti-obesity medication use after the last prescription.

Weight status documented (follow-up): Presence of clinician documentation describing post-period weight status in notes.

Max pre-index dose: Maximum recorded dose prior to the last prescription, summarized as median and interquartile range (Q1, Q3).

## Discussion

Overall, this study highlights that while patients with an active GLP-1RA prescription tend to lose significant weight, weight regain is not universally observed after the last prescription captured in the EHR. There are likely multiple factors contributing to this observation. For example, as described above, exercise counseling was documented more frequently in the non-regain cohorts. This suggests that lifestyle factors such as exercise may play an important role in modulating weight loss durability following the last GLP-1RA prescription and motivates randomized clinical trials to directly test this hypothesis. Further, some patients may have continued to have treatment exposure following the date of the last prescription, whether related to this prescription itself or medication access from another source, such as a medical spa, telehealth provider, and/or compounding pharmacy. In any case, the findings of this study can help both patients and clinicians better understand what happens in the real world after a primary care or specialist physician stops prescribing a GLP-1RA.

For multiple reasons, the data presented here is not contradictory to prior randomized controlled trials (RCTs) that have demonstrated weight regain after discontinuation of GLP-1RAs [12, 14, 19, 20]. First, this analysis focuses on weight trajectories that follow the last GLP-1RA prescription by a primary care or specialist physician, a unique “real-world index event” that has not been explored in previous studies. While our follow-up analysis on patients with confirmed discontinuation in clinical notes yielded similar findings, it is important to note that assessment of discontinuation via EHR notes has its own challenges and limitations, including incomplete documentation and automated inclusion of structured content in notes (e.g. medication lists). Second, RCTs have measured weight rebound under controlled withdrawal settings (e.g. withdrawal at a specified timepoint after therapy initiation), whereas real-world data reflect a combination of pharmacology, adaptive human behavior, selective discontinuation, flexible re-initiation patterns, and more. Indeed, real-world treatment cessation is heterogenous and often partial, staggered, or followed by re-initiation, dose tapering, lifestyle changes, or adjunctive therapies, all of which could blunt or magnify metabolic rebound physiology [8, 21]. Patients who discontinue therapy and do not restart within the following 6 months, as is the case for the cohorts analyzed here, are by definition a selected subset who likely have unique characteristics relating to treatment exposures and behavioral adaptations. The implication of this study is not that the risk of weight rebound is negligible but rather that durable response after prescription discontinuation may be achievable with an appropriate framework in place. Accordingly, it may be useful to develop systems that help clinicians better identify who can safely discontinue, who requires continued maintenance therapy, and when sustained intervention is indicated to preserve metabolic benefits.

This study has several limitations. First, it is a retrospective analysis of real-world data and is thus prone to multiple sources of confounding. For example, the inclusion criteria applied in this study (i.e. multiple prescriptions for the same medication and multiple weight measurements over a 11.5-year-period) could lead to selection bias with enrichment for patients who are more likely to have lost and kept off weight. Further, medical comorbidities and concomitant medications could impact treatment response and post-treatment weight maintenance patterns. For example, several anti-diabetic medications are known to promote weight increase (e.g. insulin) or decrease (e.g. metformin) [22]. Second, the follow-up interval is limited to 6 months, whereas prior studies have explored longer durations following treatment cessation [13, 14, 19, 23]. The shorter-interval follow-up could lead to underestimation of patients who regain weight after the last prescription, and the analyses presented here should be interpreted as early trajectories following prescription discontinuation. Indeed, in the non-regain cohorts described here, there is an upward deflection of weight at the 6-month timepoint (Fig. 2), which could in part reflect impending weight regain for at least a subset of the cohort. Conversely, re-initiation of therapy after the 6-month observation window assessed in this study would likely impact longer-term weight trajectories. Studies with longer follow-up times will be important to fully characterize weight regain dynamics. Third, there is certainly variability in treatment adherence, even leading up to the last prescription. To enrich for patients who were more likely to have had true therapy exposure, we required patients to have at least three GLP-1RA prescriptions at most 4 months apart during a one-year period in the participating health systems for inclusion in the study. However, it is likely that some of these patients missed doses or stopped taking the medication before the last documented prescription. Conversely, as mentioned previously, it is likely that some patients continued to receive the medication after their last documented prescription or after the observation window from other sources that are not captured in the EHR, such as telehealth providers or compounding pharmacies. This is an important potential source of misclassification bias that could influence the observed weight trajectories. Finally, this study did not account for social determinants of health that impact access to therapies, prescription fill rates, and treatment adherence, including but not limited to income and insurance status [24]. Future analyses are warranted to explore how these factors impact heterogeneity in weight maintenance in the post-GLP-1RA prescription interval.

In conclusion, in the study population presented here, weight loss after GLP-1RA therapy did not uniformly reverse after the last EHR-documented prescriptions. Rather, a majority of patients maintained their weight or continued to lose weight, while a smaller but important subset experienced weight regain. These findings illustrate the importance of a nuanced view of long-term weight regulation in the era of GLP-1 therapies and motivate future multimodal monitoring of patients after prescription discontinuation and/or therapy cessation which integrate biology, behavior, comorbidities, and drug dosing information. Such investigations could unlock features that can help to predict patients who are likely to show sustained benefit versus metabolic rebound.

## Conflicts of interest

KM, GV, AJV and VS are employees of nference, inc., which conducts research collaborations with various biopharmaceutical companies, including AstraZeneca, Eli Lilly and Company, and Novo Nordisk A/S, whose GLP-1 receptor agonist products (semaglutide and tirzepatide formulations) are included in this study. None of these companies, nor any other nference collaborator, funded, supported, or had any role in the independent study design, data acquisition, analysis, interpretation, manuscript preparation, or the decision to submit this work for publication. All analyses were conducted by the authors using de-identified EHR data. CMG reports consulting with nference. The authors declare no additional competing interests.

## Data Availability

This study involves the analysis of de-identified electronic health record (EHR) data via the nference nSights Federated Clinical Analytics Platform (nSights). Data shown and reported in this manuscript were extracted from this environment using an established protocol for data extraction, aimed at preserving patient privacy. The data have been de-identified pursuant to an expert determination in accordance with the HIPAA Privacy Rule. Any data beyond what is reported in the manuscript, including but not limited to the raw EHR data, cannot be shared or released due to the parameters of the expert determination to maintain the data de-identification. The corresponding author should be contacted for additional details regarding nSights.
